# Effects of Akt/mTOR/p70S6K Signaling Pathway Regulation on Neuron Remodeling Caused by Translocation Repair

**DOI:** 10.3389/fnins.2020.565870

**Published:** 2020-09-29

**Authors:** Yusong Yuan, Dongdong Li, Fei Yu, Xuejing Kang, Hailin Xu, Peixun Zhang

**Affiliations:** ^1^Department of Trauma and Orthopedics, Peking University People’s Hospital, Peking University, Beijing, China; ^2^Key Laboratory of Trauma and Neural Regeneration, Ministry of Education, Peking University, Beijing, China; ^3^National Center for Trauma Medicine, Beijing, China; ^4^Department of Orthopedics, PLA Strategic Support Force Characteristic Medical Center, Beijing, China; ^5^Diabetic Foot Treatment Center, Peking University People’s Hospital, Peking University, Beijing, China

**Keywords:** peripheral nerve injury, translocation repair, remodeling, dorsal root ganglion, signaling pathway

## Abstract

Peripheral nerve injury repair has been considered a difficult problem in the field of trauma for a long time. Conventional surgical methods are not applicable in some special types of nerve injury, prompting scholars to seek to develop more effective nerve translocation repair technologies. The purpose of this study was to explore the functional state of neurons in injured lower limbs after translocation repair, with a view to preliminarily clarify the molecular mechanisms underlying this process. Eighteen Sprague–Dawley rats were divided into the normal, tibial nerve *in situ* repair, and common peroneal nerve transposition repair tibial nerve groups. Nerve function assessment and immunohistochemical staining of neurofilament 200 (NF-200), protein kinase B (Akt), mammalian target of rapamycin (mTOR), and ribosomal protein S6 kinase (p70S6K) in the dorsal root ganglia were performed at 12 weeks after surgery. Tibial nerve function and neuroelectrophysiological analysis, osmic acid staining, muscle strength testing, and muscle fiber staining showed that the nerve translocation repair could restore the function of the recipient nerve to a certain extent; however, the repair was not as efficient as the *in situ* repair. Immunohistochemical staining showed that the translocation repair resulted in changes in the microstructure of neuronal cell bodies, and the expressions of Akt, mTOR, and p70S6K in the three dorsal root ganglia groups were significantly different (*p* < 0.05). This study demonstrates that the nerve translocation repair technology sets up a new reflex loop, with the corresponding neuroskeletal adjustments, in which, donor neurons dominate the recipient nerves. This indicates that nerve translocation repair technology can lead to neuronal remodeling and is important as a supplementary treatment for a peripheral nerve injury. Furthermore, the Akt/mTOR/p70S6K signaling pathway may be involved in the formation of the new neural reflex loop created as a result of the translocation repair.

## Introduction

Peripheral nerve injury is a common clinical condition, mainly caused by trauma such as traction, compression, cutting, and ischemia (Huckhagel et al., 2018). Peripheral nerve damage can lead to a sensory and motor dysfunction, ranging from paresthesia to limb paralysis, in the corresponding innervated area. The repair of the damaged nerves in a manner that can restore as much of the effector function as possible has, thus, always been a challenging issue in the field of trauma surgery.

The best repair method for non-defective peripheral nerve injury is tension-free nerve *in situ* suture. In case of damaged nerves with defects, the gold standard for repair is an autologous nerve transplantation; however, this often leads to the donor zone innervation. As a result, scientists began to search for new nerve injury treatments that could replace the nerve transplantation. Nerve conduit is a recent research feature. The use of different biochemical materials and the selection of different ultrastructures make the nerve conduit more closely fit to the real nerve tissue and promote the regeneration of peripheral nerves. These new types of conduits have achieved good results in laboratory researches (Qian et al., 2018a, b, 2019). However, in the face of nerve injury similar to a nerve root avulsion injury, the nerve conduits still have certain limitations. Consequently, the importance of a nerve transposition repair has been recognized. Zheng et al. (2018) used a healthy C7 brachial plexus nerve to repair a limb hemiplegia caused by cerebral palsy. Kawabata et al. (2001) used the adjacent small nerves to repair the nerve root avulsion, as well as post-ganglion injury near the foramina, resulting in the partial restoration of the injured limb movement. During the post-transposition repair process, the donor nerve dominates the damaged target organ, which means that a new and effective nerve reflex arc is established. The donor neuron is given a new functional position to control a new effector. The re-use of the damaged target organs illustrates that the new reflex arc is regulated by a mechanism that has caused a functional remodeling of the relevant nerve conduction pathway constituent cells of the nerve center, which was the donor neuron.

Using a fluorescent retrograde tracing and an enlarged model of two nerves to repair two nerves, our group previously proved that relative neurons in the spinal cord were reshaped when the effector was changed (Yu et al., 2016). However, the activation state and mechanisms of the signal transduction pathways involved in the central remodeling remained unclear.

Protein kinase B (Akt) signaling involves many pathways implicated in several biological functions, including cell proliferation, migration, and survival, and it is also closely related to tissue regeneration (Chen et al., 2013). Similarly, the mammalian target of rapamycin/ribosomal protein S6 kinase (mTOR/p70S6K) as a downstream effector of Akt activation is also a control center for cell growth and aging, especially involving the activation of the phosphatidylinositol 3-kinase/Akt signaling pathway and mTOR phosphorylation induced by Akt (Fingar and Blenis, 2004; Loewith and Hall, 2011).

This study explored the regulation of Akt/mTOR/p70S6K/toll-like receptor-4 (TLR-4) during an effector-induced neuronal structural and functional remodeling after a nerve transposition and aimed to clarify the possible mechanisms underlying this central nervous system remodeling.

## Materials and Methods

### Animals

Eighteen female Sprague–Dawley rats, aged 6 weeks and weighing 225 ± 25 g (Beijing Vital River Laboratory Animal Technology Co., Ltd., China), were randomly selected. The experimental animals were caged in a specific-pathogen free area at the Experimental Animal Center of Peking University People’s Hospital at 24 ± 2°C, 50–55% relative humidity, a 12 h light/dark cycle, and with free access to standard pellet feed and clean drinking water. This study was approved by the Ethics Committee of the People’s Hospital of Peking University, China (approval No. 2015-50). All the experimental procedures followed were in accordance with the Laboratory Animal Management Regulations of Peking University People’s Hospital.

### Surgery

All animals were randomly divided equally into three groups – the sham operation group (N group, *n* = 6), *in situ* repair group (TN-TN group, *n* = 6), and common peroneal nerve transposition repair tibial nerve group (PN-TN group, *n* = 6).

The skin of the rats was cut along the long axis of the right hind leg to expose the sciatic nerve and its branches after anesthesia using isoflurane gas (5%, 100 mL/min). For N group animals, the wound layers were closed thereafter. For TN-TN and PN-TN group animals, the tibial nerve and common peroneal nerve were cut off about 5 mm from the sciatic nerve bifurcation. The two stumps of the common peroneal nerve were ligated and sutured to the adjacent muscles in the TN-TN group. The proximal tibial nerve and the distal common peroneal nerve were ligated and sutured to the adjacent muscles in the PN-TN group. A deacetylated chitin tube (inner diameter, 0.8 mm) was used to suture the two stumps of the residual nerve in each group; the gap between the stumps was about 2 mm. Subsequently, the wound layers were sutured after irrigation with normal saline (An et al., 2015).

### Tibial Nerve Function Index (TFI)

The rats were passed through an imprinted box which the footprints could be recorded by a camera in the SPF level barrier environment at 12 weeks after the operation, and their gait patterns were recorded using a digital video camera (Canon; Tokyo, Japan). Three variables were measured: print length (PL): the longest distance of a single footprint; toe spread (TS): the distance from the 1st to 5th toe line; and the width of the middle toe (intermediary toe spread, IT): the distance from the 2nd to 4th toe line. The right foot data were used as the experimental (E) data and left foot data as the normal (N) data. The following three factors were then calculated: footprint length factor (PL factor, PLF) = (EPL–NPL)/NPL; footprint width factor (TS factor, TSF) = (ETS–NTS)/NTS; and intermediate toe width factor (IT factor, ITF) = (EIT–NIT)/NIT. The TFI was calculated using the Bain-Mackinnon-Hunter formula as follows: TFI = −37.2 (PLF) + 104.4 (TSF) + 45.6 (ITF)–8.8.

### Neuroelectrophysiological Examination

Rats were anesthetized using isoflurane gas (5.0%, 100 mL/min) after gait recording was completed. The skin was cut along the original surgical incision, the sciatic nerve was bluntly separated, and the tibialis anterior and gastrocnemius muscles were exposed. Stimulation electrodes were placed 1.0 cm apart at the distal and proximal ends of the conduit. Induction electrodes were placed on the tibialis anterior muscle. An electrophysiology instrument (Oxford Instruments Inc., Oxford, United Kingdom) was set to a rectangular pulse (duration: 0.1 ms, current: 0.09 mA, frequency: 1 Hz). The differences in t and latency between the adjacent far and near conduction times were recorded and calculated, respectively. Motor nerve conduction velocity was 0.01/t.

### Tetanic Muscle Contraction Strength

The gastrocnemius was dissected and isolated after the end of the electrophysiological test. The hind limb was fixed on a specially made holding frame, with the distal end of the gastrocnemius connected to a tension sensor such that the holding frame kept the gastrocnemius and the tension sensor aligned. The initial tension was maintained at a fixed level (0 < *F* < 0.1 N). An electrophysiological system was then used to generate an initial electric stimulation (intensity: 0.9 mA, wavelength: 0.1 ms, frequency: 1 Hz). The stimulation electric current was subsequently strengthened until the waveform of the tetanic contraction induced stopped increasing. A biomedical signal acquisition and processing system (PCLAB-UE; Beijing Microsignal Star Inc., Beijing, China) was used to record the waveform of the tetanic contraction of the gastrocnemius on both sides. The amplitudes of the waves were measured, and the ratio of the wave amplitude of the experimental side to that of the untreated normal control side was used as the overall recovery rate of muscle strength.

### Osmic Acid Staining

After the end of the tetanic muscle contraction strength test, 5 mm of nerve tissue was taken from the distal end of the suture point. The tissue samples were fixed in 4% paraformaldehyde overnight and rinsed for 8 h. Then, the samples were soaked in 1% osmic acid solution overnight in a fume hood. After 12 h of rinsing, the sample was dehydrated using an alcohol gradient. After embedding in transparent paraffin, sample slices (3 μm each) were prepared, dewaxed, subjected to alcohol gradient dehydration, and transparent xylene and neutral gum sealing. The sections were then observed under a microscope (Olympus Corporation, Tokyo, Japan); five 400–fold magnification fields of view were randomly selected for each tissue sample to count the average number of the myelinated nerve fibers using the Image Pro plus 6.0 (Media Cybernetics Inc., Rockville, MD, United States) (An et al., 2015).

### Muscle Wet Weight Weighing

The Achilles tendon was cut off to detach the gastrocnemius muscles from the distal and proximal stumps after the nerves were dissected. The muscles were weighed on an electronic balance after the blood had been wiped.

### Muscle Hematoxylin and Eosin (HE) Staining

Transverse sectioning of the muscle samples was performed for HE staining after fixing with paraformaldehyde, dehydrating with graded ethanol solutions, and embedding in paraffin wax. The cross-sections of the muscle fibers were photographed under a 400–fold magnification, and five fields were selected in the upper left, lower left, upper right, lower right, and center of the cross-section of the muscle fibers for quantification of the muscular fiber diameters in each field using Image Pro plus 6.0.

### Muscle Gomori Staining

The muscle sections were immersed in Gomori staining solution (Beijing Solarbio Science & Technology Co., Ltd., Beijing, China) for 60 min at 37°C in a sealed environment. Then, the sections were washed thrice with running water and thrice with distilled water. After being air-dried, the sections were sealed using glycerin gelatin, and the muscle fibers were subsequently viewed under a microscope.

### Dorsal Root Ganglion (DRG) HE Staining

The L5 dorsal root ganglion was excised, and the tissues were fixed, dehydrated, sliced, and dewaxed, as mentioned above. After HE staining, the sections were observed under a microscope.

### DRG Immunohistochemical Staining

Primary antibodies against neurofilament 200 (NF-200), Akt, mTOR, p70S6K, and toll like receptor 4 (TLR-4) were purchased from Abcam, Cambridge, the UK (ab82259 for NF-200, ab179463 for Akt, ab32028 for mTOR, ab32529 for p70S6K, ab22048 for TLR-4). The ganglion tissue was fixed, dehydrated, sliced, and dewaxed as described above. Pepsins were used for antigen retrieval. After washing with phosphate-buffered saline, tissues were incubated in 3% hydrogen peroxide solution for 10 min. After washing, the samples were incubated with primary antibody and then with secondary antibody, stained using 3,3′-diaminobenzidine, and observed under the microscope. Samples from the three groups were probed for the same proteins at the same time. Hematoxylin counterstain time was 3 min. Samples were subjected to a gradient alcohol dehydration and transparent xylene and neutral gum sealing; thereafter, sections were observed under a microscope. Five fields of view were randomly selected from each section and the average integrated option density (IOD) of protein positive was measured using the Image Pro plus 6.0.

### Statistical Analysis

Statistical analyses were performed using the SPSS 11.0 (SPSS Inc., Chicago, IL, United States). All data are expressed as mean ± standard deviation. Independent *t*-tests were used for two-group comparisons, such as TFI. When the data met the homogeneity of variance, the one way ANOVA was used for comparing the differences between the groups. The non-parametric test (Kruskal-Wallis k samples) was chosen when the data did not meet the homogeneity of variance which involves the pairwise comparison of every combination of group pairs. Statistical significance was defined as *P* < 0.05.

## Results

### General Observations

**FIGURE 1:**
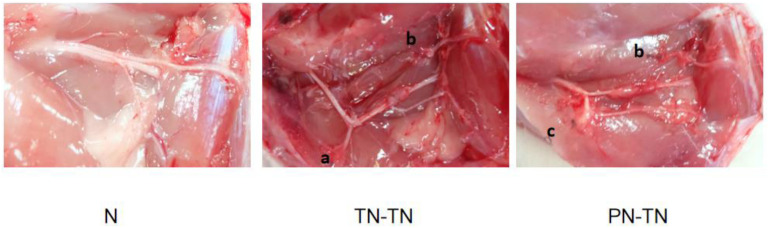
Nerve distribution. **(a)** Ligature suture point on the proximal stump of the common peroneal nerve; **(b)** ligature suture point on the distal stump of the common peroneal nerve and **(c)** ligature suture point on the proximal stump of the tibial nerve.

**FIGURE 2:**
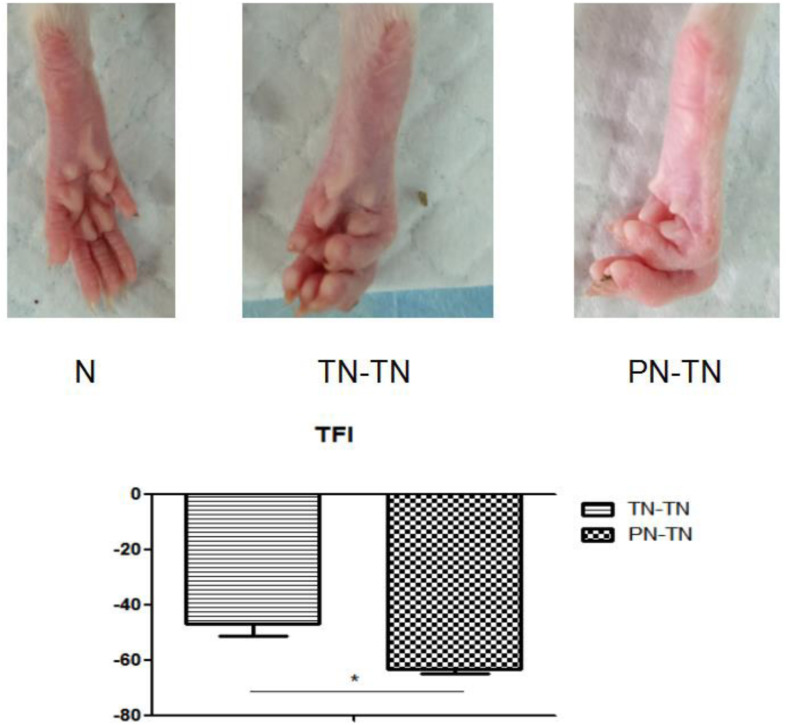
The first row shows the morphology of the right hind paw of a rat from each of the three groups. It can be seen that the toes of the rat in the N group were naturally separated with full plantar muscles. In the TN-TN group, the five-toed curling deformity was mild, and the plantar muscles were slightly atrophied. The five-toe curling deformity was obvious, and the plantar muscles were obviously atrophied in the PN-TN group. The second row shows the TFIs of PT-TN and TN-TN group. *P < 0.05.

All rats survived to the end of the experiments with bright hair. No systemic or local infections or other post-operative complications were observed. Rats in the PN-TN and TN-TN groups showed flexion deformities in the right lower limbs and atrophy of the palmar muscles, but no obvious autophagy in the toes. After the exposure to the surgical field, there was no obvious inflammatory response around the conduits. The partially absorbed conduits were found to adhere slightly to the surrounding tissue. The nerves in the conduits were observed to have grown smoothly to the terminal nerves after stripping of the incomplete cannula and local soft tissue carefully. No obvious neurofibroma formation was found at any nerve ligation or suture site (Figures 1, 2).

### TFI

The TFIs of the PN-TN and TN-TN groups were -63.37 ± 1.46 and -47.13 ± 4.07, respectively, and the difference between the two groups was statistically significant (*P* < 0.001, tested by independent *t*-tests) (Figure 2).

### Quantification of Nerve Fibers and Electrophysiological Examination

**FIGURE 3:**
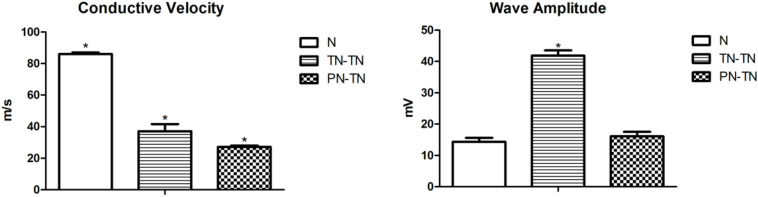
Nerve conduction velocity was significantly different between pairs, with the highest in the N group and the lowest in the PN-TN group. The TN-TN group had the largest amplitude, which was significantly higher than that of the other two groups. *P < 0.05 vs. other groups.

The results of the neural acid staining are shown in Figure 3. The number of the myelinated nerve fibers was 3878.43 ± 135.26 in the N group, 3,618.88 ± 257.76 in the TN-TN group, and 3584.48 ± 184.67 in the PN-TN group (Table 1). There was no significant difference in the number of the myelinated nerve fibers between the groups (*P* > 0.05).

**TABLE 1:** Electrophysiological examination and histological analysis results across groups.

Group	*N*	TN-TN	PN-TN
Conductive velocity (m/s)	86.02 ± 0.93*	37.12 ± 4.49*	27.14 ± 0.91*
Wave amplitude (mV)	14.41 ± 1.22	41.86 ± 1.64*	16.12 ± 1.43
Myelinated axon number	3728.43 ± 135.26	3561.88 ± 257.76	3584.48 ± 184.67

*^∗^P < 0.05 vs. other groups.*

**FIGURE 4:**
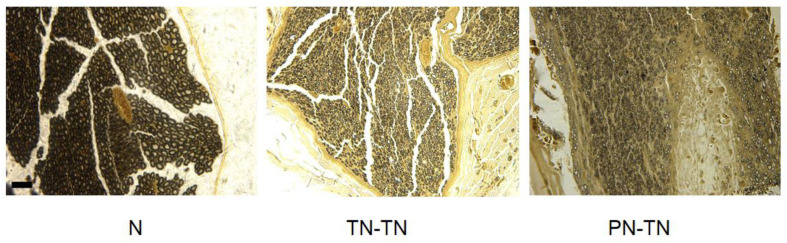
Osmic acid staining. The myelinated nerve fibers in the N group were evenly distributed and the axon area was significantly larger than those of the other two groups. Both the TN-TN group and the PN-TN group had different degrees of fiber invasion and unevenly distributed myelinated nerve fibers. Scale bar: 25 μm.

The nerve conduction velocity and compound action potential amplitude data are shown in Table 1. The nerve conduction velocity in the surgical groups was worse than that in the normal group [*P* = 0.001, tested by the non-parametric test (Kruskal-Wallis k samples)]. The nerve conduction velocity in the TN-TN group was significantly higher than that in the PN-TN group (*p* < 0.001, tested by one way ANOVA). The composite action potential amplitude of the TN-TN group was significantly higher than those of the other two groups (*P* < 0.001 vs. N group and *P* < 0.001 vs. PN-TN group, tested by one way ANOVA); however, there was no significant difference in this regard between the PN-TN group and the N group (*P* = 0.093, tested by one way ANOVA) (Figure 4).

### Gastrocnemius Wet Weight and Strength

**FIGURE 5:**
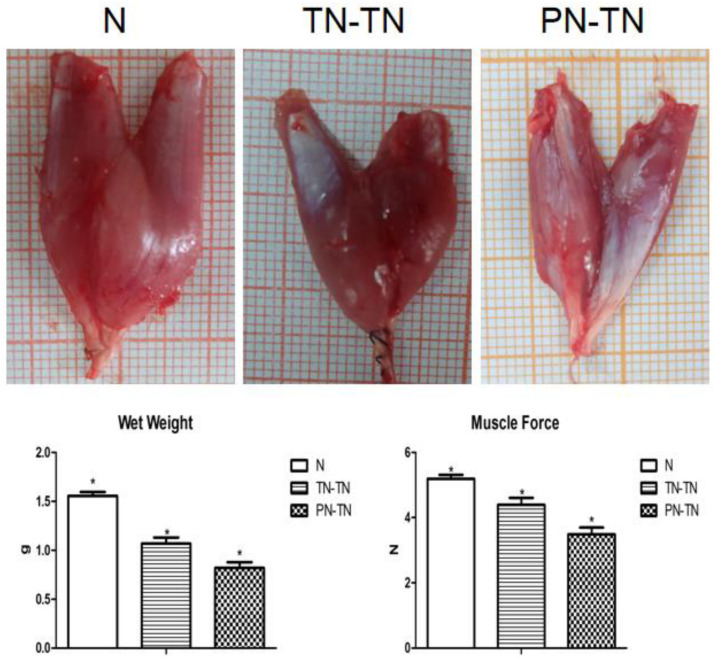
The first row shows the appearance of the right gastrocnemius muscle in each group. The gastrocnemius muscles in the N group were large while gastrocnemius atrophy was significant in the PT-TN group. The second row shows the muscle wet weights and strengths. Consistent with the appearance of the muscles, the muscle status of the N group was the best and that of the PN-TN group was the worst. *P < 0.05 vs. other groups.

The gastrocnemius muscle weight of the surgery group was significantly lower than that of the normal group (*P* < 0.001, tested by one way ANOVA) (Table 2). The nutritional status of the gastrocnemius muscle in the TN-TN group was significantly better than that in the PN-TN group (Figure 5).

**TABLE 2:** Gastrocnemius muscle characteristics across groups.

Group	*N*	TN-TN	PN-TN
Wet weight (g)	1.56 ± 0.04*	1.07 ± 0.06*	0.82 ± 0.06*
Muscle force (*N*)	5.19 ± 0.12*	4.39 ± 0.21*	3.49 ± 0.21*
Muscular fiber diameter (μm)	34.54 ± 7.25*	23.33 ± 3.58*	18.67 ± 2.94*

*P < 0.05 vs. other groups.

The muscle forces of the three groups showed the same trend as that of the wet weight (Table 2). Muscle strength was the highest in the N group and the lowest in the PN-TN group. There was a significant difference in muscle strength between the three groups (*P* < 0.001, tested by one way ANOVA).

### Muscle Fibers Morphology

**FIGURE 6:**
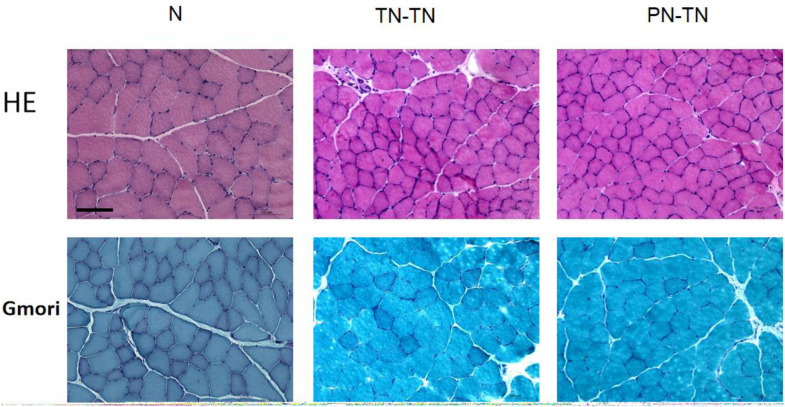
Morphology of muscle fibers. The muscle fibers in group N were distinctly angular and closely arranged. In the TN-TN and PN-TN groups, oval-shaped muscle fibers could be seen and the gap between the muscle fibers was widened. The muscle fiber diameter of the N group was significantly larger than those of the other two groups. The Gomori staining was normal and there were no signs of mitochondrial disease. Scale bar: 100 μm.

The muscle fiber diameters were 34.54 ± 7.25, 23.33 ± 3.58, and 18.67 ± 2.94 μm for the N, TN-TN, and PN-TN groups, respectively; the differences between the three groups were statistically significant (*P* < 0.001, tested by one way ANOVA). Based on the results of the Gomori staining, no mitochondrial lesions were observed in the muscle fibers in any of the three groups (Figure 6).

### DRG Morphology

**FIGURE 7:**
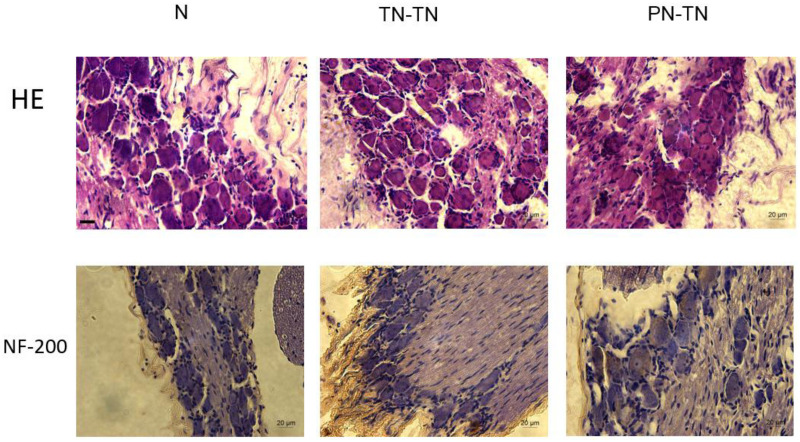
DRG morphology and immunohistochemical staining. Yellow-brown staining represents NF-200 positive cells. Scale bar: 20 μm.

The morphology and distribution of the dorsal root ganglion neurons were not significantly different among the three groups; the neurons in all three groups were clustered and near the spinal cord. The expression of NF-200 in the PN-TN group was significantly higher than that in the other two groups. The expression of NF-200 in the TN-TN group was slightly higher than that in the sham operation group, but the difference was not statistically significant (Figure 7).

### Expression of Akt/mTOR/p70S6K

**FIGURE 8:**
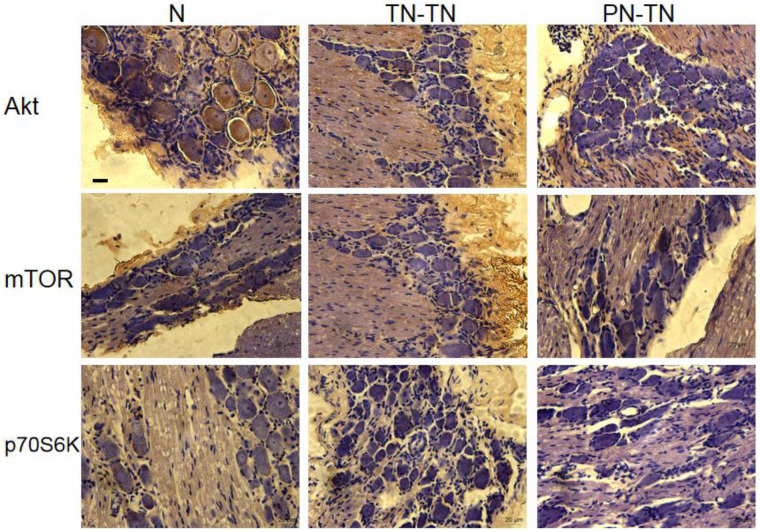
Expression of Akt/mTOR/p70S6K in the DRG. Yellow-brown staining represents protein expression, with a more intense color representing higher expression levels. Scale bar: 20 μm.

The expression levels of AKT and p70S6K were highest in the normal group, followed by those in the TN-TN group; expression was the lowest in the PN-TN group. The expression of mTOR was the lowest in the normal group and slightly higher in the TN-TN group, while the PN-TN group showed the highest expression. The differences in the expression levels of these three proteins between the three groups were statistically significant (Figure 8 and Table 3).

**TABLE 3:** The expression of Akt, mTOR, p70S6K in each group.

	AKT (*10^–2^)	mTOR (*10^–2^)	p70S6K (*10^–2^)
N	15.28 ± 0.78	6.45 ± 0.53	10.68 ± 0.61
TN-TN	7.37 ± 0.56	8.38 ± 0.45	5.89 ± 0.40
PN-TN	2.52 ± 0.36	11.60 ± 0.85	0.83 ± 0.04

*The value is IOD of each protein.*

## Discussion

The repair of a nerve damage has always been a key issue for neuroscientists around the world; however, the development of more effective treatments for limb paralysis and dysfunction due to a nerve damage has been stagnant (Palmisano et al., 2019). At present, the number of patients with dysfunction after a peripheral nerve injury in China is close to 20 million, and it is increasing at a rate of nearly 2 million per year (Fowler et al., 2015). In case of some special types of nerve injury, such as a nerve root laceration and avulsion, traditional surgical techniques such as tension-free nerve adventitia implantation, autologous nerve transplantation, and nerve cannula have limited efficacy (Kubota et al., 2011; Rebowe et al., 2018; Sun et al., 2019). Zhang et al. creatively proposed C7 nerve transposition from the healthy side to repair the paralyzed upper limb and gained nice effects (Jiang et al., 2018; Zheng et al., 2018). The use of an adjacent nerve transposition to repair paralysis and the use of the brachial plexus nerve of the affected limb of children for similar purposes has also had encouraging results. The recent popularization and application of the Shore reflex arc has also been beneficial for some patients with urinary incontinence (Lam Van Ba et al., 2018). In our laboratory, we found that when the effector changes were dominated by the neuron clusters in the spinal cord, the function and anatomical distribution of its neurons changed, which meant that the transposition repair method built a new, functional reflection loop (Yu et al., 2016).

In our earlier studies, we also found that, during the process of nerve injury and repair, the functional state of effectors induces the remodeling of the central nervous system. The survival status of the neuronal cell body determines the regeneration potential of the nerve after a peripheral nerve injury and subsequent surgical repair of the injured nerve. Furthermore, the specific selection of the distal and proximal ends of the nerve fiber and the multi-factor regulation of the regeneration state determines the nerve regeneration process. The central nervous system will recognize the changes of the peripheral effectors and regulate their function when the regenerated nerve fiber establishes a new connection with a well-preserved peripheral effector. At the same time, the peripheral effector also induces the corresponding structural and functional remodeling of neurons at all levels. Thus, the nerve function after repair reflected the original function of the repaired peripheral effector.

By enabling the transmission of the internal and external signals, the protein kinase signal cascade plays a very important role in the regulation of many cellular processes, including cell survival, proliferation, differentiation, growth cessation, and apoptosis. With the deepening of our understanding of the protein signaling mechanisms and the exploration of the role of protein signaling cascades under normal physiological conditions, the role of protein signaling cascades under pathological conditions has attracted attention. Extracellular signals, such as mechanical forces transmitted by trauma, stimulate the protein kinase cascade, which can lead to the induction and activation of the transcription factors that can regulate gene expression related to cell growth and apoptosis, thereby affecting the damaged cell and tissue repair and plasticity. The post-injury persistent activation properties of some protein kinases suggest the existence of critical time windows for the treatment of various nervous system injuries (Neary, 2005).

Akt is at the center of a cellular signaling network that plays an important role in key biological functions and cellular processes (Manning and Toker, 2017). mTOR is an important downstream signaling molecule in the Akt pathway and can be activated through phosphorylation by AKT. It is generally believed that mTOR exerts its role through phosphorylation to activate two downstream signaling molecules: the eukaryotic promoter 4E binding protein 1 and P70S6K (Burnett et al., 1998).

Akt is also specifically involved in the neural processes such as neuronal development (Jaworski et al., 2005; Kumar et al., 2005), differentiation (Otaegi et al., 2006; Lim et al., 2007), survival, axonal growth (Sango et al., 2008; Toth et al., 2008), and mediation of the synaptic plasticity (Wang et al., 2003; Horwood et al., 2006). In addition, some studies (Dudek et al., 1997; Owada et al., 1997; Brunet et al., 2001) have also shown that the post-optic nerve and hypoglossal nerve injury increase in Akt expression is related to both the anti-apoptotic effects and axon regeneration. After a brain injury (Namikawa et al., 2000; Noshita et al., 2002; Kretz et al., 2005) and spinal cord injury (Hung et al., 2007; Shioda et al., 2007, 2008; Chen et al., 2008), its enhanced expression has neuroprotective effects and is beneficial for the recovery of neural function. As a central regulator of cell growth, cell cycle progression, survival, and differentiation, mTOR also plays an important role in the physiological and pathological processes of the nervous system (Sakurai et al., 2003; Yu et al., 2005), and its possible role in central nervous system trauma has also attracted considerable attention. Studies have shown that mTOR is involved in the regulation and control of the size of neurons in the dentate gyrus and hippocampus (Hay and Sonenberg, 2004). As an important meeting point for multiple signaling pathways, mTOR has been shown to control the neuronal development (Kwon et al., 2006; Swiech et al., 2008), differentiation (Jaworski and Sheng, 2006; Muir et al., 2019), and regeneration by regulating global or local protein synthesis. Several studies (Dash et al., 2006; Bekinschtein et al., 2007; Parsons et al., 2010) have also reported that mTOR activation is related to memory formation and spatial memory. By regulating the downstream component p70S6K, mTOR has also been shown to upregulate the expression of long-term potentiating effector proteins required for synaptic connections (Tang et al., 2002).

In this study, we transposed the common peroneal nerve to repair the tibial nerve and compared the results with those of *in situ* repair of the tibial nerve as a control to explore the possible mechanisms underlying the successful establishment of an effective reflex arc by the transposition repair. In PN-TN group, the continuity of the nerve had been maintained and well repaired with regard to both the microstructure and the macrostructure. Furthermore, its electrical signal transmission ability had been restored, resulting in the innervation of the tibialis anterior muscle; certain systolic functions of this muscle had been restored, and rats in the PN-TN group could exercise the dorsiflexion function. This suggested that the effector changes induced neurons to undergo a functional remodeling. However, in the process of transposition repair, there was still a certain gap in the recovery of neural function compared with that in *in situ* nerved repair. Between the three groups, there were significant differences in the nerve conduction velocity, compound action potential amplitude, myelinated nerve fiber number, myelin sheath thickness, and muscle wet weight. The repair group was worse than the sham operation group, while the PN-TN group was worse than the TN-TN group.

Interestingly, after the immunohistochemical staining for NF-200 in the dorsal root ganglia, it was found that the expression levels of N group, TN-TN group, and PN-TN group increased gradually with obvious differences. It was suggested that the effector could induce the neuron to undergo a structural remodeling to adapt to the new reflex loop from a neuroskeletal structure perspective. This change might be related to the regeneration of axonal buds during the nerve repair. Peripheral nerves possess the ability of axillary bud regeneration during the regeneration process, which means that a proximal axon can generate several axillary buds and connect to the distal nerve stump. This theory provides the anatomical foundation for small nerve translocation to repair large nerves. Even with *in situ* nerve adventitia suture, more distal nerve fibers are more enlarged than the proximal nerve fibers in the early stage of nerve regeneration. However, during the long-term recovery, axillary bud trimming will occur, and the enlargement ratio will gradually approach one (Jianping et al., 2012; Barbe et al., 2017). Therefore, the proximal nerves in both the PN-TN and TN-TN groups developed lateral axis buds when the distal access channel was fixed but were translocated relative to the corresponding neurons of the original tibial nerve. Compared to the neurons in the spinal cord corresponding to the original tibial nerve, the number of axillary buds corresponding to the transfigured and repaired peroneal neurons would be higher, which meant a more complicated axoplasm transport process and a complicated neural skeleton. Therefore, the expression of NF-200, a neuroskeletal proteins, in the DRG of the PN-TN group was higher than that of the TN-TN group.

Akt/mTOR/p70S6K is a protease cascade signaling pathway related to cell proliferation, with phosphorylation as the activation mode. Significant differences in Akt, mTOR, and p70S6K expression was observed among the three groups in this study. Compared with that in the other two groups, the expression of Akt and p70S6K was lower in the PN-TN group, while the expression of mTOR was higher. It is suggested that in the process of an effector–induced neuronal structural and functional remodeling, different repair methods ultimately affect the neuron’s survival state; thus translocation repair results in a special, new, and effective reflection arc different from that in *in situ* repair.

The limitation of this study was that only the spontaneous changes of the Akt/mTOR/p70S6K signaling pathway during an effector – induced neuronal remodeling were investigated, and the remodeling process involving these three small molecule inhibitors were not clarified clearly. More complex regulatory mechanisms involving the three proteins were not explored. However, it was the first time to explore the molecular mechanism of a spinal cord remodeling caused by the lower limbs nerve transposition repair. We will do more in-depth research on this basis in the future.

In summary, this study proved that the translocation repair is also an effective nerve repair method. This kind of repair could promote the structural and functional remodeling of the central neurons and result in the formation of a new reflex arc. Furthermore, the Akt/mTOR/p70S6K signaling pathway might play an important role in the central nervous system remodeling.

## Data Availability Statement

All datasets presented in this study are included in the article/supplementary material.

## Ethics Statement

The animal study was reviewed and approved by the Ethics Committee of the People’s Hospital of Peking University, China.

## Author Contributions

HX and PZ designed and supported the study, and reviewed the manuscript. YY and DL performed the experiments and analyzed the data. FY and XK assisted with the experiments. All authors contributed to the article and approved the submitted version.

## Conflict of Interest

The authors declare that the research was conducted in the absence of any commercial or financial relationships that could be construed as a potential conflict of interest.
